# Optimization of Enzymatic Protein Hydrolysate from Mung Bean (*Vigna radiata* L.), and Its Functional Properties

**DOI:** 10.3390/foods14142459

**Published:** 2025-07-13

**Authors:** Kanokwan Promjeen, Suphat Phongthai, Kanjana Singh, Worrapob Chaisan, Peeraporn Pakakaew, Somdet Srichairatanakool, Rajnibhas Sukeaw Samakradhamrongthai, Niramon Utama-ang

**Affiliations:** 1 Division of Product Development Technology, Faculty of Agro-Industry, Chiang Mai University, Chiang Mai 50100, Thailand; kanokwan_pr@cmu.ac.th (K.P.); kanjana_singh@hotmail.co.th (K.S.); rajnibhas.s@cmu.ac.th (R.S.S.); 2 Division of Food Science and Technology, Faculty of Agro-Industry, Chiang Mai University, Chiang Mai 50100, Thailand; suphat.phongthai@cmu.ac.th (S.P.); peeraporn1994@gmail.com (P.P.); 3 Cluster of High Value Products from Thai Rice and Plants for Health, Chiang Mai University, Chiang Mai 50100, Thailand; worrapob.ch@gmail.com; 4 Department of Biochemistry, Faculty of Medicine, Chiang Mai University, Chiang Mai 50200, Thailand; somdet.s@cmu.ac.th; 5 ThaiBrit FoodBridge: Collaboration Between Thailand and UK Sensory and Consumer Science Networks for Sustainable Agri-Food Supply, Faculty of Agro-Industry, Chiang Mai University, Chiang Mai 50100, Thailand

**Keywords:** mung bean, protein hydrolysate, mung bean peptides, functional properties, alcalase enzyme, optimization

## Abstract

Mung bean is a rich protein source, but its native form has limited solubility and functionality for food applications. As a promising agro-based crop, mung bean offers a sustainable alternative to traditional protein sources, especially in regions with limited access to resources. This study optimized mung bean protein hydrolysate (MBPH) production using response surface methodology (RSM), investigating the effects of alcalase concentration (2–7%) and hydrolysis time (2–7 h) on its physicochemical and functional properties. The results showed that an alcalase concentration of 5.88% and a hydrolysis duration of 3.56 h were the optimal conditions, resulting in a degree of hydrolysis of approximately 33.09%. Under these conditions, MBPH contained 79.33 ± 0.62% protein and a molecular weight distribution of 45.57% and 47.29% at 1.1–10 kDa and <10 kDa, respectively. Additionally, MBPH exhibited strong antioxidant activity, improved foam capacity, and enhanced solubility, making it a valuable ingredient for sustainable food production and promoting equitable access to nutritious functional ingredients.

## 1. Introduction

Mung bean (*Vigna radiata* L.) is a widely cultivated legume known for its high protein content and well-balanced amino acid composition, making it a promising candidate for functional food applications [1]. While mung beans have been a staple in global diets for centuries, particularly in Asian countries, consuming them in whole form can be challenging due to the presence of large protein molecules and anti-nutritional factors, which may cause digestive dis-comfort [2]. Consequently, mung beans are often processed into more digestible food products. However, the native functional properties of mung bean proteins, such as solubility, emulsification, and antioxidant activity, remain suboptimal, necessitating targeted modifications to enhance their applicability in food systems. Research has shown that mung bean peptides (MBPs) or mung bean protein hydrolysates (MBPHs), derived from the enzymatic hydrolysis of mung bean proteins, exhibit various physiological benefits, including hypoglycemic and hypolipidemic effects, antihypertensive properties, and antioxidant and anticancer activities [3]. Additionally, MBPs exhibit greater digestibility and absorption in the human body than intact mung bean proteins [3,4]. These advantages suggest promising applications in functional beverages, meal replacements, and plant-based emulsified foods. However, further work is needed to address certain limitations related to product stability, sensory characteristics, and scalability in food formulations. The growing demand for sustainable and functional plant-based proteins highlights the importance of optimizing hydrolysate properties to meet industry needs.

The production of protein hydrolysates has gained significant attention in research due to its cost-effectiveness and potential to mitigate environmental impacts [5]. Enzymatic hydrolysis is a widely employed technique in food science to modify protein structure and improve solubility, emulsification, foaming properties, and antioxidant activity [6]. The controlled cleavage of peptide bonds by specific proteases generates hydrolysates with desirable physicochemical and bioactive properties, expanding their potential applications in functional foods and nutraceuticals [7]. Alcalase, a serine endopeptidase, has been extensively utilized for protein hydrolysis due to its broad specificity and efficiency in producing bioactive peptides. Many studies have used alcalase to produce MBPHs. Zheng et al. [8] used 2% (*w*/*w*) alcalase to hydrolyze mung bean for 5 h to produce MBPHs, while Karami et al. [9] used 7% (*w*/*w*) alcalase for 7 h. Liu et al. [10] employed 3000 U/g of substrate for 4 h, and alcalase was added to the solution to a final concentration of 5000 U/g of mung bean protein for 2 h [2]. Chen et al. [11] used 4.9% (*w*/*v*) alcalase for 4 h to prepare MBPH. Based on these reports of alcalase concentrations between 2% and 7% and hydrolysis times of 2 to 7 h, this study selected slightly broader ranges (1–10% enzyme concentration and 3–9 h hydrolysis time) to ensure the comprehensive coverage for experimental design. Since hydrolysis conditions, including enzyme concentration and reaction time, critically affect the functional and nutritional properties of protein hydrolysates [12], this research aimed to optimize the conditions for hydrolyzing MBPHs using alcalase through response surface methodology. This study evaluated the effects of enzyme concentration and hydrolysis time on yield, protein content, antioxidant activity, and key functional properties such as water and oil absorption, emulsification, and foaming.

The optimal conditions for MBPH production were then used to evaluate its physicochemical properties, including amino acid composition and Fourier-transform infrared (FTIR) spectroscopy, in comparison to mung bean protein isolate (MBPI).

## 2. Materials and Methods

### 2.1. Sample Preparation

#### 2.1.1. Sample Source and Preparation

A clean and dehulled mung bean (*Vigna radiata* L.) was purchased from Thanya Farm Co., Ltd. (Nonthaburi, Thailand). It was soaked for 6 h at room temperature and milled with water in a 1:1 ratio using a wet grinder (PHILIPS HR2068, Philips Electronics (Thailand) Ltd.) (Bangkok, Thailand). The sample was then dried at 50 °C for 24 h, ground into flour, and passed through a 100-mesh sieve [10]. Mung bean flour was defatted in a hexane solution at a 1:3 (*w*/*v*) ratio, using a stirrer at 500 rpm for 40 min [13]. After that, the samples were dried in a fume hood to minimize the hexane content [14] in the flour for 24 h and stored at 4 °C until use.

#### 2.1.2. Mung Bean Protein Isolate (MBPI) Preparation

The MBPI was prepared following the method of Liu et al. [10], with minor modifications. Defatted mung bean flour (5% *w*/*v*) was mixed with distilled water. The suspension was stirred until it reached 40 °C, then adjusted to pH 9.5 using a 1 M NaOH solution, and maintained at 40 °C for 1 h. After centrifugation at 4000 rpm for 10 min (Allegra 64R Centrifuge, Beckman Coulter Inc., Indianapolis, IN, USA), the supernatant was collected and its pH adjusted to 4.6 with 1.0 M HCl. The solution was then centrifuged again. The resulting precipitate was collected, dispersed in distilled water, and adjusted to pH 7.0 using 1 M NaOH. Samples were frozen at −80 °C for 12 h, then lyophilized at −55 °C under vacuum for 48 h. The resulting mung bean protein extract was stored at room temperature until further use.

### 2.2. Mung Bean Protein Hydrolysate (MBPH) Preparation

#### 2.2.1. Experimental Design

A two-level factorial design was applied to identify significant factors and optimal conditions for hydrolyzing mung bean protein. The factors included alcalase (Sigma Chemical Company, St. Louis, MO, USA) concentrations at 1%, 5%, and 10% (*v*/*w*) and hydrolysis times of 3, 6, and 9 h. Protein hydrolysate preparation followed the design table (Table 1) generated by Design-Expert software 6.0.10, with all factors randomized across 9 experimental runs. First, 3 g of MBPI was dissolved in 100 mL of distilled water (3% *w*/*v*). The solution was then hydrolyzed using alcalase at enzyme-to-substrate ratios of 1:100, 5:100, and 10:100 (*v*/*w*). The pH was adjusted to 8.5 and maintained using 1 M NaOH, and the reaction was carried out at 55 °C. The hydrolysis process followed the experimental setup in Table 1. Enzyme inactivation was performed by placing the reaction tube in a 95 °C water bath for 15 min [13], followed by cooling to room temperature. The cooled mixture was centrifuged at 10,000× *g* for 20 min at 4 °C to obtain the supernatant. The pH was adjusted to 7.0 prior to freeze-drying, and the resulting powder was stored until use.

Responses were measured for hydrolysate yield, protein content, antioxidant activity (DPPH and ABTS), water absorption, oil absorption, foam capacity, foam stability, emulsifying activity index (EAI), emulsion stability index (ESI), protein solubility at pH 3, 5, 7, and 9, and degree of hydrolysis (DH). SPSS software version 17.0 was used to perform analysis of variance (ANOVA) on the experimental results, with a significance level set at 95%.

#### 2.2.2. Analysis of Mung Bean Protein Hydrolysate

1. Yield

The yield of the mung bean protein hydrolysate was determined according to the method described by Romadhoni et al. [15], using the following Equation (1):

Yield (%) = [weight of hydrolysate product (g)/weight of raw material (g)] × 100(1)

2. Protein content

The protein content was assessed by determining the total nitrogen using a protein analyzer (FOSS, Kjeltec, Denmark). The protein concentration was calculated using a Kjeldahl factor of 6.25 [16] and expressed as % dw (dry weight).

3. DPPH radical scavenging activity

The DPPH radical scavenging capacity of the mung bean protein hydrolysate was determined according to the method of Utama-Ang et al. [17]. A solution of 0.1 mM DPPH in 95% ethanol (1 mL) was combined with 0.25 mL of the sample solution (5 mg/mL), then incubated in the dark at a room temperature for 30 min. Absorbance was recorded at 517 nm using a spectrophotometer (Libra S22, Biochrom, Cambridge, MA, USA). Trolox was used to prepare the standard curve, and the scavenging activity was expressed in mmol Trolox/g.

4. ABTS radical scavenging activity

The ABTS radical scavenging activity of the sample was assessed following the method described by Utama-Ang et al. [17]. Briefly, the ABTS radical was produced by mixing 7 mM ABTS and 2.45 mM potassium persulfate in a 1:1 ratio, then incubating the mixture in the dark at room temperature for 12 h. Before use, the ABTS solution was diluted with 5 mM phosphate buffer (pH 7.4) until the absorbance at 734 nm reached 0.70 ± 0.02. Equal volumes of sample and ABTS radical solution were then mixed and incubated at room temperature for 10 min. The absorbance was subsequently recorded at 734 nm. Trolox was used to prepare the standard curve, and the scavenging activity was expressed in mmol Trolox/g.

5. Water absorption capacity

The water absorption capacity (WAC) was determined according to the method of Piornos et al. [18]. A 0.5 g sample was mixed with 5 mL of distilled water and allowed to stand for 30 min. The slurry was then centrifuged at 6000 rpm for 10 min, and the unabsorbed water was decanted and weighed. The water absorption value was calculated using Equation (2) and expressed as the mass (g) of water per unit mass (g) of protein.

WAC (g water/g protein) = [W_0_ − W_s_]/W_0_ (2)

where W_0_ is the initial mass of water used (g) and W_s_ is the mass of water recovered in the supernatant (g).

6. Oil absorption capacity

The oil absorption capacity (OAC) was determined following the method of Piornos et al. [18]. A 0.5 g sample was mixed with 5 mL of refined soybean oil and allowed to stand for 30 min. The slurry was then centrifuged at 6000 rpm for 10 min, and the unabsorbed oil was decanted and weighed. The oil absorption value was calculated using Equation (3) and expressed as the mass (g) of oil per unit mass (g) of protein.

OAC (g oil/g protein) = [W_0_ − W_s_]/W_0_ (3)

where W_0_ is the initial mass of oil used (g) and W_s_ is the mass of unabsorbed oil recovered in the supernatant (g).

7. Foam capacity and foam stability

A 50 mL aliquot of mung bean protein hydrolysate (20 mg/mL) was transferred into a graduated cylinder and homogenized at 10,000 rpm for 1 min. Volumes before and after homogenization were measured using a 100 mL graduated cylinder [19]. Foaming capacity and foam stability were calculated using Equations (4) and (5), respectively.

Foaming capacity (%) = [(V_2_ − V_1_)/V_1_] × 100 (4)

Foam stability (%) = [(V_3_ − V_2_)/V_2_] × 100 (5)

where V_1_ is the initial volume before homogenization, V_2_ is the volume after homogenization, and V_3_ is the foam volume remaining 10 min post-homogenization at room temperature.

8. Emulsion activity index (EAI) and Emulsion stability index (ESI)

First, 30 mL of MBPH at a 5% (*w*/*v*) concentration was mixed with 10 mL of soybean oil. The mixture was homogenized at 10,000 rpm for 2 min. Then, 200 μL of the emulsion sample was mixed with 25 mL of 0.1% sodium dodecyl sulfate [20]. The absorbance of the resulting solution was measured at 500 nm using a UV–vis spectrophotometer. Equations (6) and (7) were used to calculate the EAI in m^2^/g and the ESI in minutes [21]:

EAI (m^2^/g) = (2 × 2.303 × A_0_ = 0:25 × Protein concentration) (6)

ESI (min) = (A_0_ × Δt/ΔA) (7)

where A_0_ = absorbance at 0 min, A_10_ = absorbance at 10 min, ΔA = A_0_ − A_10_ and Δt = 10 min.

9. Protein solubility

A total of 0.5 g of MBPH and 0.3 g of albumin were dissolved in 100 mL of distilled water. The pH of each sample was adjusted to 3.0, 5.0, 7.0, and 9.0 using 1 M HCl solution, followed by vortex. The supernatant was then collected by centrifugation at 4000 rpm for 10 min and diluted five-fold, as described by Tsumura et al. [22]. The total protein concentration was measured using the Lowry method and compared with a bovine serum albumin standard [23].

10. Degree of hydrolysis (DH)

The DH of MBPH was determined using a modified trichloroacetic acid (TCA) method [24]. Approximately 2 g of protein hydrolysate was dissolved in 20 mL of distilled water, followed by the addition of 20 mL of 20% (*w*/*v*) TCA to precipitate proteins. The mixture was incubated at 4 °C for 30 min and centrifuged at 7000 rpm for 15 min to obtain the TCA-soluble fraction. The nitrogen content of the hydrolysate and the supernatant from the TCA-treated sample were analyzed using the Kjeldahl method. The DH was calculated as follows in Equation (8):

DH (%) = [Soluble N in TCA 10% (*w*/*v*) × 100]/Total N in the sample (8)

### 2.3. Comparison the Characterization of MBPI and MBPH

#### 2.3.1. Molecular Weight (MW) Analysis by Size Exclusion Chromatography

The molecular weight distribution of MBPI and MBPH was analyzed via size exclusion chromatography (SEC), as outlined by Zhang et al. [25]. A Shimadzu HPLC system (Kyoto, Japan) equipped with a UV–VIS detector set at 280 nm and an SRT-C SEC-300 column (5 µm, 7.8 × 300 mm, Sepax Technologies, Newark, DE, USA) was used. The column was equilibrated and operated isocratically with 0.1 M sodium phosphate buffer (pH 7.0) at a flow rate of 1.0 mL/min, maintaining the temperature at 30 °C. Samples were filtered through 0.45 µm membranes prior to the injection of 15 µL. The calibration curve was prepared by plotting the logarithm of molecular weights of the standard proteins against their elution times. The standards covered a range of 15–600 kDa and consisted of bovine thyroglobulin (~670 kDa), bovine gamma globulins (150 kDa), chicken egg albumin grade VI (44.3 kDa), ribonuclease A from bovine pancreas (13.7 kDa), along with p-aminobenzoic acid (pABA) as the low-molecular-weight marker. The area under the chromatogram was integrated to estimate the relative size distribution.

#### 2.3.2. Fourier-Transform Infrared Spectroscopy (FTIR)

FTIR was employed to examine the secondary structure of MBPI and MBPH following the method outlined by Xie et al. [26], with slight modifications. A small portion of MBPI and MBPH (0.001 g) will be combined with 0.1 g of potassium bromide, then ground and pressed into 1 mm slices under an infrared lamp. Each sample will be scanned over the range of 900 to 4000 cm^−1^, with 32 scans taken and averaged.

#### 2.3.3. Amino Acid Analysis

Seventeen amino acids were analyzed using the post-column reaction method adapted from Masuda et al. [27]. The system was equipped with a Shim-pack Amino-Na column (100 mm × 6.0 mm I.D., 5 µm; P/N: 228-18837-91, Shimadzu, Kyoto, Japan) and a Prominence RF-20A fluorescence detector (Shimadzu, Japan). Three Na-type mobile phases (A, B, and C) were prepared. Sodium citrate buffers at pH 3.23 and 10.0 were used for phases A and B, respectively, while phase C consisted of a 0.2 M aqueous sodium hydroxide solution. The pre-column derivatization of amino acids was carried out using phthaldialdehyde (OPA) and N-acetylcysteine as reagents. The chromatographic setup involved maintaining the column oven at 60 °C, operating at a flow rate of 0.4 mL/min, and injecting a 10 µL sample.

### 2.4. Statistical Analysis

All experiments were performed in triplicate, and the data represent the mean values. An analysis of variance (ANOVA) was conducted, followed by Duncan’s new multiple range test to determine significant differences, using SPSS 17.0 software (SPSS Inc., Chicago, IL, USA). A *p*-value of≤ 0.05 was considered indicative of significant differences between the samples. The response surface analysis utilized a generalized second-order polynomial model, which is represented in Equation (9).

(9) $$Y=\beta_{0}+\beta_{1}X_{1}+\beta_{2}X_{2}+\beta_{12}X_{1}X_{2}+\beta_{11}X_{1}^{2}+\beta_{22}X_{2}^{2}$$

where *Y* represents the response, *X*_1_, *X*_2_ are the independent variables or studied factors; *β*_0_ is the constant of the equation; *β*_1_, *β*_2_ are the linear equation coefficients; *β*_12_ is the interaction equation coefficients; and *β*_11_, *β*_22_ are the quadratic equation coefficients.

## 3. Results and Discussion

### 3.1. Effect of Enzyme Concentration and Hydrolysis Time on the MBPH

#### 3.1.1. Product Yield

The enzymatic hydrolysis of the protein substrate was conducted under varying conditions, with enzyme concentrations of 1%, 5%, and 10% (*w*/*v*) and hydrolysis times of 3, 6, and 9 h. The yield ranged from 6.54 ± 1.10% to 13.29 ± 2.03% (Table 2). All experiments showed no significant differences in yield (*p* > 0.05). Increasing the enzyme concentration beyond 1% did not lead to proportional improvements in yield, suggesting possible substrate limitation or product inhibition.

#### 3.1.2. Protein Content Analysis

The protein content of the hydrolysates exhibited significant variation (*p* < 0.05) across different treatment conditions, ranging from 71.42 ± 0.50% to 81.32 ± 0.01% dry weight (Table 2). A clear inverse correlation was observed between enzyme concentration and protein content. Samples treated with 1% enzyme concentration consistently showed higher protein content (79.32–81.32%) compared to those treated with 10% enzyme concentration (71.42–72.65%). The highest protein content (81.32 ± 0.01%) was achieved at 1% enzyme concentration and 9 h of hydrolysis (Experiment 3), suggesting that mild hydrolysis conditions better preserve protein structural integrity, resulting in higher detectable protein content. At higher enzyme concentrations, extensive hydrolysis may lead to the formation of very small peptides and free amino acids, which are not efficiently detected by standard protein quantification methods [28], resulting in lower apparent protein content.

#### 3.1.3. DPPH Radical Scavenging Activity

DPPH radical scavenging activity ranged from 2.10 ± 0.83 to 7.29 ± 0.78 mmol Trolox/g (Table 2). A positive correlation was observed between DPPH activity and enzyme concentration (Table 3), with higher enzyme concentrations resulting in increased DPPH values. The highest DPPH value, 7.29 ± 0.78 mmol Trolox/g, was observed at 10% enzyme concentration and 9 h of hydrolysis. This may be due to more extensive hydrolysis that releases peptides with enhanced antioxidant properties [29], which is consistent with the findings of Kusumah et al. [30], who suggest that increasing enzyme concentration and extending the hydrolysis time enhance radical scavenging activity. Several studies have demonstrated that increasing the enzyme concentration and extending hydrolysis time can significantly enhance the radical scavenging activity of protein hydrolysates [11,13]. This enhancement is primarily attributed to structural modifications, reduced molecular weight, and the increased exposure of functional groups.

WAC refers to water absorption capacity, OAC denotes oil absorption capacity, ESI represents the emulsion stability index, and DH stands for the degree of hydrolysis.

Molecular weight peptides (<3 kDa) typically exhibit superior antioxidant activity due to improved diffusion and interaction with free radicals [1]. Furthermore, the release of specific amino acid residues such as histidine, tyrosine, methionine, and tryptophan has been closely associated with radical scavenging activity, given their ability to donate protons or electrons [2]. Additionally, extended hydrolysis increases the number of exposed functional groups, including amino (–NH_2_), hydroxyl (–OH), and carboxyl (–COOH) groups. These groups play a critical role in neutralizing free radicals through hydrogen atom transfer and metal ion chelation mechanisms [3]. In summary, the improved antioxidant properties observed with higher enzyme concentrations and longer hydrolysis times are mainly due to the generation of bioactive peptides with favorable size, structure, and reactive group availability.

#### 3.1.4. ABTS Radical Scavenging Activity

ABTS radical scavenging activity ranged from 134.99 ± 6.45 to 163.70 ± 2.51 μmol Trolox/100 g (Table 2). The highest activity was observed in samples treated with 10% enzyme concentration at 3 h (163.70 ± 2.51 μmol Trolox/100 g), while the lowest activity was found in samples treated with 5% enzyme concentration at 9 h (134.99 ± 6.45 μmol Trolox/100 g). Unlike DPPH activity, ABTS radical scavenging showed a more complex relationship with hydrolysis conditions, suggesting different mechanisms of action for these two antioxidant pathways. The reduction of ABTS free radicals in the presence of MBPH suggests that it effectively generates a blend of peptides and amino acids capable of neutralizing ABTS radicals, likely by pairing with the radicals’ single electrons [9].

#### 3.1.5. Water Absorption Capacity (WAC)

The WAC exhibited significant variations (*p* < 0.05) across different hydrolysis conditions, ranging from 1.58 ± 0.35 to 4.80 ± 0.34 g water/g protein (Table 2). A positive correlation was observed between the enzyme concentration and WAC, with the highest value (4.80 ± 0.34 g water/g protein) achieved at 10% enzyme concentration and 6 h of hydrolysis (Experiment 8). The increase in WAC with a higher enzyme concentration may be attributed to the exposure of additional hydrophilic groups during protein hydrolysis, which creates more water-binding sites [28]. The WAC of proteins is primarily attributed to the presence of -COOH, -NH_3_, and -OH groups in their structure. The probable absence of these polar groups in the hydrolysates reduces their interaction with water, which may explain the decreased WAC observed when a low level of alcalase was used for hydrolysis [31]. The unfolding of the quaternary structure by enzyme hydrolysis exposed hidden hydrophilic groups, which may enhance interaction with water molecules and contribute to the increased WAC [32].

#### 3.1.6. Oil Absorption Capacity (OAC)

The OAC demonstrated an inverse relationship with the enzyme concentration, ranging from 1.37 ± 0.31 to 3.61 ± 0.35 g oil/g protein (Table 2). The highest OAC (3.61 ± 0.35 g oil/g protein) was observed at 1% enzyme concentration and 9 h hydrolysis time (Experiment 3), while the lowest (1.37 ± 0.31 g oil/g protein) occurred at 10% enzyme concentration and 6 h (Experiment 8). This trend suggests that extensive hydrolysis may reduce the protein’s ability to bind oil, possibly due to the breakdown of the hydrophobic domains responsible for oil interaction [33].

#### 3.1.7. Foam Capacity

The foam capacity ranged from 97.50 ± 3.54% to 24.00 ± 1.41% (Table 2), showing a significant decrease with increasing enzyme concentration and hydrolysis time. The highest foam capacity was observed under mild hydrolysis conditions at 1% alcalase and 3 h, while the lowest was recorded under intensive hydrolysis conditions at 10% enzyme and 9 h. This inverse relationship indicates that extensive hydrolysis reduces the protein’s ability to form stable interfacial films, which are essential for foam formation. Alcalase-driven hydrolysis generated a certain quantity of low-molar-mass peptides that contributed to foaming capacity due to their flexible molecular structure, low molar mass, and amphiphilic characteristics [34].

#### 3.1.8. Foam Stability

Foam stability followed a similar trend to foam capacity with values ranging from 43.67 ± 0.47% to 8.67 ± 0.47% (Table 2). The highest stability was recorded in samples treated with 1% enzyme concentration for 3 h (Experiment 1), while the lowest was found at the 10% enzyme concentration for 9 h (Experiment 9). The significant reduction in foam stability (*p* < 0.05) at higher enzyme concentrations suggests that extensive hydrolysis may produce shorter peptides, reducing the ability to maintain stable foam structures. This effect might result from strong hydrophobic interactions and the exchange between free sulfhydryl groups and disulfide bonds, which encourages peptide aggregation, stabilizing their conformation and forming a thicker, stronger liquid film around bubbles compared to the previously formed short-chain peptides [35].

#### 3.1.9. Emulsifying Activity Index (EAI)

The EAI values ranged from 4.10 ± 0.81 to 8.03 ± 0.05 m^2^/g, showing significant variation (*p* < 0.05) with the hydrolysis conditions (Table 2). The highest EAI was observed at 1% enzyme concentration and 3 h of hydrolysis (Experiment 1), while the lowest was recorded at 10% enzyme concentration and 6 h (Experiment 8). These results suggest that a mild degree of hydrolysis may be optimal for maintaining the emulsifying ability, possibly due to the retention of the amphiphilic peptide sequences necessary for emulsion formation [36].

#### 3.1.10. Emulsion Stability Index (ESI)

The ESI values ranged from 70.79 ± 2.73% to 93.65 ± 0.33%, showing significant differences across the treatments (*p* < 0.05), as presented in Table 2. The highest stability was observed at 1% enzyme concentration and 3 h of hydrolysis (Experiment 1), whereas lower stability was generally associated with higher enzyme concentrations and longer hydrolysis times. This trend suggests that extensive hydrolysis may reduce the capacity of protein to maintain stable emulsions [37].

#### 3.1.11. Solubility

The effects of enzyme concentration and hydrolysis time on protein solubility across a pH range of 3 to 9 are crucial because solubility significantly influences the functional properties of protein hydrolysates in food applications [38]. As shown in Table 2, solubility varied with pH and hydrolysis conditions, increasing from as low as 51.30% at pH 3 to complete solubility (100%) at pH 9 for all samples. Solubility increased with pH, reaching 100% at pH 9, suggesting enhanced solubility under alkaline conditions due to increased protein–water interactions [28]. At a pH of 7, solubility ranged from 87.05% to 97.27%, indicating improved protein solubility under neutral conditions. Notably, all protein samples achieved complete solubility (100.00 ± 0.00%) at a pH of 9, regardless of the enzyme concentration or hydrolysis time, suggesting that alkaline conditions promote maximum protein solubility independently of hydrolysis parameters. In an alkaline environment, proteins carry a strong net negative charge, resulting in greater interaction with the aqueous surroundings and, consequently, enhanced solubility [28].

#### 3.1.12. Degree of Hydrolysis (DH)

The DH values of MBPH ranged from 28.48% to 41.51%. The lowest DH value of 28.48% was observed with 1% enzyme concentration and 3 h of hydrolysis, while the highest DH value of 41.51% was recorded with 10% enzyme concentration and 9 h of hydrolysis. These variations in DH indicate the influence of processing conditions on the extent of protein hydrolysis, with specific conditions favoring higher DH levels [39]. As the DH increased, the peptide chains shortened, and the molecular weight distribution narrowed due to the cleavage of the peptide bonds [40]. The DH results also correspond to the solubility profiles of the hydrolysates at different pH levels. Higher DH values are generally associated with higher protein solubility, particularly at neutral and alkaline pH levels (pH 7 and pH 9), suggesting an enhancement in protein solubility due to increased hydrolysis.

#### 3.1.13. Regression Analysis

**Figure 1:**
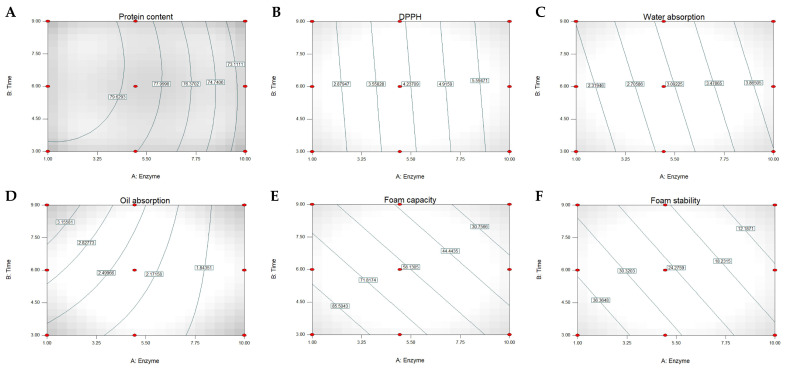

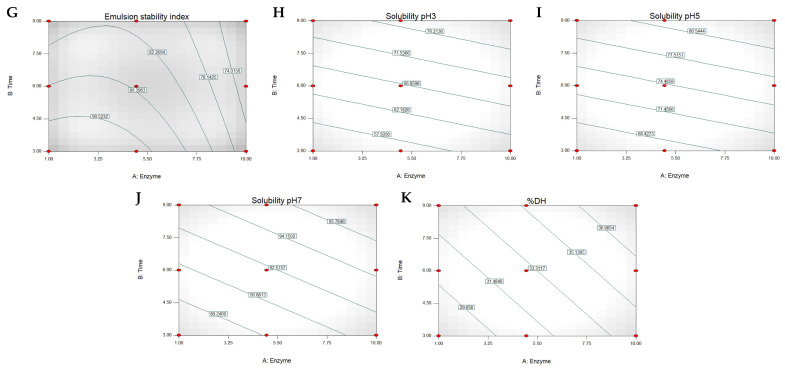
Response surface plots showing the effect of enzyme and time hydrolysate on protein content (**A**), DPPH (**B**), water absorption (**C**), oil absorption (**D**), foam capacity (**E**), foam stability (**F**), emulsion stability index (**G**), solubility at pH 3, 5, and 7 (**H**–**J**), and degree of hydrolysis (**K**).

Based on the data in Table 2, multiple regression analysis was performed to evaluate the relationships between enzyme concentration, hydrolysis time, and the various functional properties of the mung bean protein hydrolysates. The results found that 10 out of 14 responses showed statistical significance (*p* < 0.05), as shown in Table 3. The response surface plots were used to illustrate the relationships between enzyme concentration, hydrolysis time, and the significance variables (Figure 1).

The protein content model demonstrated the highest coefficient of determination (*R*^2^ = 0.9990, *p* < 0.0001), represented by a quadratic equation with both linear and interaction terms. The model indicated that protein content was positively influenced by both enzyme concentration (0.42) and time (1.28), while showing negative quadratic effects for both factors (−0.10 × Enzyme^2^ and −0.08 × Time^2^) and a slight negative interaction effect (−0.046 × Enzyme × Time). This pattern aligns with the contour plot surface of the protein content as presented in Figure 1A. The contour lines showed a gradual decrease in protein content moving toward both higher enzyme concentrations and longer hydrolysis times, as evidenced by the values decreasing from 79.63% in the central region to 73.11% at the edges of the experimental space.

Antioxidant activity, measured by DPPH radical scavenging capacity, showed a simpler linear relationship (*R*^2^ = 0.7358, *p* = 0.0185) with both enzyme concentration (0.43) and time (0.04) having positive effects. The DPPH values increased consistently with both enzyme concentration and hydrolysis time (Figure 1B). Similarly, water absorption exhibited a linear positive correlation with both factors (*R*^2^ = 0.6849, *p* = 0.0313). The water absorption contour plot displayed nearly parallel diagonal lines (Figure 1C). The water absorption values increased progressively from approximately 2.32 to 3.82 g/g as both the enzyme concentration and hydrolysis time increased. Oil absorption demonstrated a strong fit (*R*^2^ = 0.9641, *p* = 0.0005) with a negative interaction between the enzyme concentration and time (−0.02 × Enzyme × Time). Unlike water absorption, the oil absorption capacity demonstrated nonlinear behavior (Figure 1D), with the highest values (>3.25 g/g) observed at low enzyme concentrations (<3.25%) and extended hydrolysis times (>7.5 h). The 2D pattern consistency with the negative interaction term (−0.02 × enzyme × time) in the regression equation.

Both foam capacity (*R*^2^ = 0.8911, *p* = 0.0013) and foam stability (*R*^2^ = 0.8808, *p* = 0.0017) showed negative linear relationships with enzyme concentration and time, suggesting that increased hydrolysis led to decreased foaming properties. This data was consistent with the contour surface plot of foam capacity (Figure 1E) and foam stability (Figure 1F). The emulsion stability index exhibited a complex relationship (*R*^2^ = 0.9863, *p* = 0.0054) with both quadratic and interaction terms. The contour plot for emulsion stability index revealed a nonlinear relationship (Figure 1G), with maximum ESI values (>82) observed at moderate enzyme concentrations (5.5–7.75) and higher reaction times (7.5–9.0 h).

Protein solubility at different pH values (3, 5, and 7) showed strong linear relationships with both factors. The models demonstrated increasing baseline values with increasing pH (41.38, 57.93, and 84.21 for pH 3, 5, and 7, respectively), while maintaining positive coefficients for both enzyme concentration and time. The strongest correlation was observed at pH 7 (*R*^2^ = 0.9396, *p* = 0.0002). The contour plots of solubility at pH 3 (Figure 1H), pH 5 (Figure 1I), pH 7 (Figure 1J) also showed a linear relationship between enzyme concentration and hydrolysis time. The contour lines indicate a consistent effect of both variables on solubility throughout the experimental range. The model of DH yielded an *R*^2^ value of 0.6583, indicating that approximately 65.83% of the variability in DH can be explained by the combined effects of enzyme concentration and hydrolysis time. The *p*-value of 0.0399 suggests that the model is statistically significant at a 5% significance level, confirming the relevance of the enzyme concentration and hydrolysis time in influencing DH. The 2D plot indicates a positive linear relationship between both factors and DH, with an increase in either enzyme concentration or time resulting in an increase in DH (Figure 1K).

From the results, the regression models (Table 3) and the contour plots (Figure 1) provide valuable insights for optimizing hydrolysis conditions to achieve the desired functional properties in mung bean protein hydrolysates.

#### 3.1.14. Optimization and Verification of MBPH

**Figure 2:**
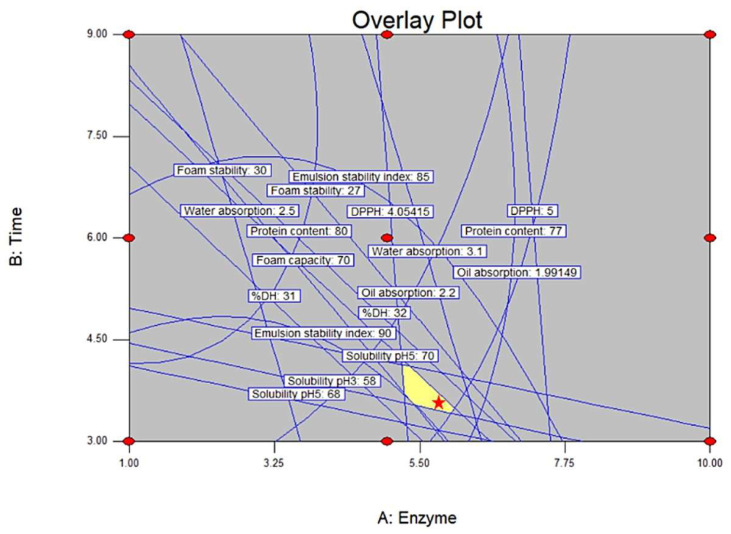
The overlay plot shows the optimal conditions for the mung bean protein hydrolysate. The yellow area with a red star indicates the best condition region.

The optimal conditions for enhancing the properties of MBPH were achieved with an alcalase concentration of 5.88% and a hydrolysis time of 3.56 h (Figure 2). Under these conditions, MBPH exhibited the following properties: protein content of 79.33 ± 0.62%, DPPH of 4.98 ± 0.37 mmol Trolox/g, water absorption of 3.04 ± 1.54 g water/g protein, oil absorption of 2.13 ± 0.83 g oil/g protein, foam capacity of 71.43 ± 1.77%, foam stability of 27.80 ± 0.88%, ESI of 85.72 ± 2.31%, solubility of 57.17 ± 1.51% at pH 3, 67.25 ± 1.71% at pH 5, 93.56 ± 1.16% at pH 7, and DH of 33.09 ± 1.19%. A 10% prediction error was observed in almost all parameters, except for DPPH, which showed a 15% error (Table 4). However, these deviations are still considered acceptable.

The protein content of the peptides from this study was close to that reported by Zheng et al. [8], who used 2% (*w*/*w*) alcalase to hydrolyze mung bean for 5 h and obtained a protein content of 83.19%. In contrast, the optimal conditions for producing MBPH as determined by Sonklin et al. [29] were 15% (*w*/*w*) bromelain with a hydrolysis duration of 12 h [29]. Karami et al. performed hydrolysis individually using alcalase under conditions of 55 °C, pH 8, for 7 h at 7% enzyme concentration, and flavourzyme under conditions of 50 °C, pH 6, for 5 h at 7% enzyme concentration [9].

### 3.2. Comparisons of Molecular Weight Distribution, Secondary Structure, and Amino Acid Profiles Between MBPI and MBPH

#### 3.2.1. Molecular Weight

The molecular weight distribution of MBPI after enzymatic hydrolysis under optimal conditions was analyzed using size exclusion chromatography, as shown in Table 5. The results showed distinct differences between MBPI and MBPH. MBPI contained a high proportion of large molecular weight fractions, with 20.32 ± 2.87% >100 kDa and 59.68 ± 0.08% in the 11–100 kDa range. In contrast, MBPH mainly consisted of smaller peptides, with 3.11 ± 0.01% in the 1.1–10 kDa range and 4.03 ± 0.01% below 1 kDa. This indicates that alcalase effectively cleaves peptide bonds, breaking down larger protein molecules into smaller peptides and free amino acids, which could contribute to the improved functional and bioactive properties of the hydrolysate [41]. In addition, low molecular weight peptide fractions have been reported to exhibit higher antioxidant activities compared to the crude MBPH [29].

#### 3.2.2. Protein Secondary Structure

**Figure 3:**
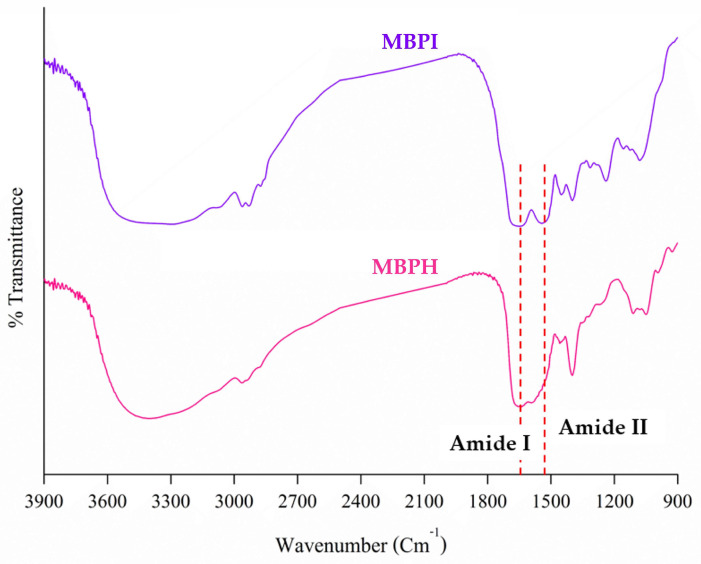
FTIR spectra of mung bean protein isolate (MBPI, purple line) and mung bean protein hydrolysate (MBPH, pink line).

The FTIR spectra of MBPI and MBPH revealed characteristic changes in the protein’s secondary structure (Figure 3). Both samples exhibited typical amide bands, with notable peaks corresponding to Amide I (approximately 1650 cm^−1^) and Amide II (approximately 1550 cm^−1^). The complex secondary structures of Amide I are due to the stretching of C=O and the bending of N-H bonds [42]. The spectra showed broad absorption bands in the region of 3300–3500 cm^−1^, attributable to N-H stretching vibrations, and 2900–3000 cm^−1^, corresponding to C-H stretching. The peak intensity and width of MBPI were greater than those of MBPH within the 3000–3500 cm^−1^ range, indicating the presence of strong hydrogen bonds among MBPI molecules, which were partially disrupted by alcalase [10,43]. The spectral ranges of 1650–1660 cm^−1^, 1600–1640 cm^−1^, 1640–1650 cm^−1^, and 1660–1700 cm^−1^ corresponded to α-helix, β-sheets, random coils, and β-turns, respectively [43].

Analysis of the secondary structure revealed significant structural alterations between MBPI and MBPH (Table 5). MBPI contained higher proportions of α-helix (18.87 ± 0.14%) and β-sheet (29.62 ± 0.44%), whereas MBPH showed reduced levels of α-helix (15.48 ± 0.17%) and β-sheet (12.31 ± 0.07%). In contrast, MBPH exhibited a pronounced increase in β-turns (52.68 ± 1.25%) compared to MBPI (32.17 ± 0.32%). Both samples had comparable levels of random coils, with MBPI showing 19.35 ± 0.62% and MBPH 19.52 ± 0.42%. These findings suggest that enzymatic hydrolysis alters the protein’s secondary structure, leading to a more flexible and unfolded state in MBPH. These structural changes, particularly the decrease in ordered structures (α-helix and β-sheet) and increase in β-turn content, suggest substantial protein unfolding and reorganization during the hydrolysis process [10,40]. The reduction in α-helix and β-sheet structures was due to the breaking of most hydrogen bonds in the proteins by alcalase [10]. Likewise, the structure of protein hydrolysates from mung bean, black bean, and sweet potato becomes looser following enzymatic hydrolysis [10,40,44]. This structural transformation may contribute to the altered functional properties and potentially enhanced bioactivity of the hydrolysate compared to the native protein isolate.

#### 3.2.3. Amino Acid Content

The distribution of hydrophilic and hydrophobic amino acids in MBPI and MBPH reveals significant variations, as presented in Table 5, reflecting the impact of enzymatic hydrolysis on protein composition and structure.

The changes in amino acid composition may influence the functional properties and potential bioactive characteristics of the hydrolysate compared to the original protein isolate [10]. MBPH exhibited a higher overall proportion of hydrophobic amino acids compared to MBPI, reflecting the exposure of buried hydrophobic regions during protein hydrolysis [29]. Conversely, hydrophilic amino acids dominated in MBPI, maintaining its solubility and hydrophilicity. The higher concentration of hydrophobic amino acids in MBPH suggests improved emulsifying and surface-active properties, which are critical for its functional application in food systems [10]. In contrast, the predominance of hydrophilic amino acids in MBPI may enhance its water solubility, favoring its use in aqueous formulations. The significant increase in histidine in MBPH highlights the selective release of certain amino acids during hydrolysis, which could enhance bioactivity [29]. Similarly, elevated levels of leucine and methionine in MBPH may contribute to its nutritional profile.

MBPI and MBPH differed significantly in molecular weight distribution, secondary structure, and amino acid composition, which contributed to the enhanced functional properties of MBPH. As shown in Table 5, MBPI predominantly contained larger proteins (>100 kDa: 20.32%, 11–100 kDa: 59.68%), whereas MBPH was abundant in smaller peptides (1.1–10 kDa: 3.11%, <1 kDa: 4.03%). This distribution reflects effective hydrolysis by alcalase. Moreover, secondary structure analysis revealed that MBPI retained ordered structures typical of native proteins, with α-helix and β-sheet contents of 18.87% and 29.62%, respectively. In contrast, MBPH showed increased β-turns (52.68%) accompanied by reductions in α-helix and β-sheet content, indicating protein unfolding. This structural alteration likely contributed to the improved water and oil absorption capacities of MBPH.

Despite the promising results observed in this study, certain limitations should be acknowledged. Firstly, although the optimized hydrolysis conditions improved the functional properties of MBPH, factors such as batch-to-batch variability, protein source heterogeneity, and enzyme specificity could influence the reproducibility of results. Moreover, sensory properties—including flavor and odor, which are critical for consumer acceptance—were not evaluated and require further investigation. In terms of practical applications, MBPH exhibits desirable solubility and emulsifying properties, suggesting potential for use in plant-based beverages, meal replacements, and emulsified food systems. However, challenges related to product stability under varying pH and temperature conditions, as well as the scalability of enzymatic hydrolysis processes at the industrial level, must be addressed before commercial implementation.

## 4. Conclusions

This study successfully optimized the hydrolysis conditions for MBPH production, achieving an ideal Alcalase concentration of 5.88% and a hydrolysis time of 3.56 h. The optimized MBPH exhibited significantly improved solubility, emulsifying capacity, and antioxidant activity, highlighting its potential as a valuable functional ingredient for plant-based food systems. Additionally, a comparative analysis with MBPI confirmed structural modifications that contributed to the enhanced bioactivity of the hydrolysate. Importantly, the use of a moderate enzyme concentration demonstrated practical feasibility, as it produced high-quality hydrolysate without the need for excessive enzyme usage. Furthermore, earlier results suggest that even lower enzyme concentrations, such as 1%, may still offer acceptable functional properties, supporting the feasibility of enzyme reduction depending on specific industrial objectives. This strategy may help reduce production costs and enhance scalability in industrial applications. Future work should focus on incorporating MBPH into real food matrices, evaluating sensory attributes, and further optimizing enzyme usage to enhance commercial viability.

## Figures and Tables

**Table 1:** Design of three-level factorial experiment of the mung bean protein hydrolysate.

Experimental Number	Factors	
	Enzyme Concentration (%)	Hydrolysate Time (h)
1	1	3
2	1	6
3	1	9
4	5	3
5	5	6
6	5	9
7	10	3
8	10	6
9	10	9

**Table 2:** The physicochemical properties of mung bean protein hydrolysates obtained through a three-level factorial analysis.

Experiments	Yield (%) ^ns^	Protein Content (%dw)	DPPH (mmol Trolox/g)	ABTS (µmol Trolox/100 g)	WAC (g Water/g Protein)	OAC (g Oil/g Protein)	Foam Capacity (%)	Foam Stability (%)	EAI (m^2^/g)	Solubility (%) at				**DH (%) ^ns^**
										pH 3	pH 5	pH 7	pH 9 ^ns^	
1	6.54 ± 1.10	79.32 ± 0.15 ^b^	3.63 ± 0.36 ^d,e^	138.92 ± 2.10 ^d,e^	1.66 ± 0.35 ^d^	2.42 ± 0.35 ^b,c^	97.50 ± 3.54 ^a^	43.67 ± 0.47 ^a^	8.03 ± 0.05 ^a^	51.30 ± 2.86 ^c^	63.57 ± 3.72 ^d^	87.05 ± 1.89 ^c^	100.00 ± 0.00	28.48 ± 0.64
2	13.29 ± 2.03	80.77 ± 0.88 ^a^	2.28 ± 0.13 ^e^	141.85 ± 1.62 ^d,e^	1.58 ± 0.35 ^d^	2.86 ± 0.34 ^a,b^	88.50 ± 2.12 ^b^	37.22 ± 1.01 ^b^	6.89 ± 0.04 ^b^	68.42 ± 1.37 ^b^	75.05 ± 2.47 ^b,c^	91.89 ± 3.60 ^a,b,c^	100.00 ± 0.00	32.34 ± 4.37
3	8.86 ± 0.35	81.32 ± 0.01 ^a^	2.10 ± 0.83 ^e^	150.41 ± 2.27 ^c^	3.03 ± 0.35 ^b,c^	3.61 ± 0.35 ^a^	69.50 ± 0.71 ^d^	34.50 ± 0.71 ^c^	5.91 ± 0.17 ^c,d^	73.38 ± 2.96 ^b^	83.74 ± 3.76 ^a^	93.53 ± 2.64 ^a,b^	100.00 ± 0.00	32.87 ± 2.71
4	9.95 ± 0.21	78.15 ± 0.32 ^c^	2.55 ± 0.86 ^e^	154.52 ± 1.26 ^b,c^	2.68 ± 0.34 ^c^	2.05 ± 0.35 ^b,c,d^	79.00 ± 1.41 ^c^	30.63 ± 0.88 ^d^	5.10 ± 0.11 ^d^	54.94 ± 1.89 ^c^	67.41 ± 0.72 ^c,d^	88.23 ± 0.64 ^b,c^	100.00 ± 0.00	29.44 ± 5.42
5	9.40 ± 0.43	79.10 ± 0.13 ^b,c^	4.75 ± 0.33 ^c,d^	135.85 ± 6.54 ^e^	3.68 ± 0.35 ^b^	2.40 ± 0.35 ^b,c^	43.50 ± 2.12 ^f^	19.50 ± 0.71 ^e^	5.15 ± 0.57 ^d^	66.80 ± 2.58 ^b^	71.09 ± 0.10 ^c,d^	92.46 ± 2.97 ^a,b,c^	100.00 ± 0.00	34.73 ± 3.98
6	11.07 ± 1.92	78.79 ± 0.81 ^b,c^	2.80 ± 0.72 ^e^	134.99 ± 6.45 ^e^	2.82 ± 0.35 ^c^	2.42 ± 0.35 ^b,c^	40.50 ± 0.71 ^f^	11.56 ± 0.63 ^g^	6.75 ± 0.24 ^b,c^	73.27 ± 6.61 ^b^	75.02 ± 2.75 ^b,c^	94.96 ± 3.22 ^a^	100.00 ± 0.00	30.33 ± 1.66
7	10.72 ± 1.85	71.91 ± 0.27 ^d,e^	5.38 ± 0.97 ^b,c^	163.70 ± 2.51 ^a^	3.79 ± 0.30 ^b^	1.69 ± 0.35 ^c,d^	63.00 ± 2.83 ^e^	20.71 ± 1.01 ^e^	5.02 ± 0.62 ^d,e^	57.90 ± 2.24 ^c^	69.85 ± 2.87 ^c,d^	92.61 ± 0.66 ^a,b,c^	100.00 ± 0.00	32.05 ± 7.06
8	9.07 ± 1.27	72.65 ± 0.19 ^d^	6.71 ± 0.74 ^a,b^	146.58 ± 1.81 ^c,d^	4.80 ± 0.34 ^a^	1.37 ± 0.31 ^d^	25.50 ± 0.71 ^g^	15.83 ± 1.18 ^f^	4.10 ± 0.81 ^e^	72.86 ± 1.79 ^b^	79.82 ± 2.85 ^a,b^	94.00 ± 0.39 ^a^	100.00 ± 0.00	38.31 ± 3.54
9	9.64 ± 2.53	71.42 ± 0.50 ^e^	7.29 ± 0.78 ^a^	161.20 ± 1.37 ^a,b^	3.47 ± 0.34 ^b,c^	1.68 ± 0.33 ^c,d^	24.00 ± 1.41 ^g^	8.67 ± 0.47 ^h^	4.98 ± 0.13 ^d,e^	81.76 ± 1.14 ^a^	84.13 ± 7.24 ^a^	97.27 ± 2.06 ^a^	100.00 ± 0.00	41.51 ± 0.75

Note: Values are presented as the mean ± SD. Superscript letters (a–h) within the same column represent statistically significant differences at *p* ≤ 0.05. ns indicates no significant difference (*p* > 0.05). dw, dry weight.

**Table 3:** Analysis of variance (ANOVA) for the experimental response based on the significant variables.

Responses		Regression Equation	*R* ^2^	*p*-Value
Protein content (%dw)		75.95 + 0.42 × Enzyme + 1.28 × Time − 0.10 × Enzyme^2^ − 0.08 × Time^2^ − 0.046 × Enzyme × Time	0.9990	0.0001
DPPH (mmol Trolox/g)		1.67 + 0.43 × Enzyme + 0.04 × Time	0.7358	0.0185
WAC (g water/g protein)		1.52 + 0.21 × Enzyme + 0.07 × Time	0.6849	0.0313
OAC (g oil/g protein)		1.88 − 0.02 × Enzyme + 0.20 × Time − 0.02 × Enzyme × Time	0.9641	0.0005
Foam capacity (%)		121.99 − 5.23 × Enzyme − 5.86 × Time	0.8911	0.0013
Foam stability (%)		51.66 − 2.54 × Enzyme − 2.24 × Time	0.8808	0.0017
ESI (%)		103.26 + 0.67 × Enzyme − 3.47 × Time − 0.29 × Enzyme^2^ + 0.08 × Time^2^ + 0.16 × Enzyme × Time	0.9863	0.0054
Solubility (%) at	pH 3	41.38 + 0.74 × Enzyme + 3.57 × Time	0.9337	0.0003
	pH 5	57.93 + 0.46 × Enzyme + 2.34 × Time	0.7828	0.0103
	pH 7	84.21 + 0.43 × Enzyme + 0.99 × Time	0.93956	0.0002
DH (%)		24.58 + 0.69 × Enzyme + 0.84 × Time	0.6583	0.0399

**Table 4:** Comparison of the predicted and experimental values of mung bean protein hydrolysate.

Responses		Predict Value	Actual Value	Error (%)
Protein content (%dw)		77.59	79.33 ± 0.62	2.24
DPPH (mmol Trolox/g)		4.31	4.98 ± 0.37	15.55
WAC (g water/g protein)		3.01	3.04 ± 1.54	1.00
OAC (g oil/g protein)		2.01	2.13 ± 0.83	5.97
Foam capacity (%)		70.47	71.43 ± 1.77	1.36
Foam stability (%)		28.78	27.80 ± 0.88	3.41
ESI (%)		89.03	85.72 ± 2.31	3.72
Solubility (%) at	pH 3	58.42	57.17 ± 1.51	2.14
	pH 5	68.96	67.25 ± 1.71	2.48
	pH 7	90.25	93.56 ± 1.16	3.67
DH (%)		31.66	33.09 ± 1.19	4.52

**Table 5:** Comparison of the molecular weight distribution, secondary structure, and amino acid content between mung bean protein isolate (MBPI) and mung bean protein hydrolysate (MBPH).

		MBPI	MBPH
Molecular weight (kDa)	>100	20.32 ± 2.87 ^a^	3.11 ± 0.01 ^b^
	11–100	59.68 ± 0.08 ^a^	4.03 ± 0.01 ^b^
	1.1–10	15.03 ± 2.74 ^b^	45.57 ± 0.04 ^a^
	<1	4.27 ± 0.02 ^b^	47.29 ± 0.06 ^a^
Secondary structure (%)	α-helix	18.87 ± 0.14 ^a^	15.48 ± 0.17 ^b^
	β-sheet	29.62 ± 0.44 ^a^	12.31 ± 0.07 ^b^
	β-turn	32.17 ± 0.32 ^b^	52.68 ± 1.25 ^a^
	Random coil ^ns^	19.35 ± 0.62	19.52 ± 0.42
Hydrophilic amino acid	Arginine	10.90 ± 1.27 ^a^	3.00 ± 0.0 4 ^b^
	Glutamic acid	13.55 ± 0.19 ^b^	73.70 ± 0.13 ^a^
	Serine	0.86 ± 0.12 ^b^	2.97 ± 0.01 ^a^
	Threonine	1.65 ± 0.02 ^ns^	1.67 ± 0.10 ^ns^
	Histidine	1.92 ± 0.27 ^b^	31.53 ± 0.03 ^a^
	Lysine ^ns^	3.53 ± 0.26	3.72 ± 0.01
	Aspartic acid	3.40 ± 0.03 ^b^	17.65 ± 0.01 ^a^
Hydrophobic amino acid	Alanine and cysteine	11.60 ± 1.14 ^a^	9.01 ± 0.02 ^b^
	Glycine	1.09 ± 0.03 ^a^	0.23 ± 0.01 ^b^
	Leucine	2.28 ± 0.16 ^b^	18.91 ± 0.07 ^a^
	Valine	2.08 ± 0.07 ^b^	3.62 ± 0.01 ^a^
	Isoleucine	0.73 ± 0.26 ^b^	1.65 ± 0.01 ^a^
	Phenylalanine	9.80 ± 0.16 ^b^	34.58 ± 0.26 ^a^
	Proline	1.41 ± 0.02 ^a^	0.05 ± 0.01 ^b^
	Methionine	0.16 ± 0.03 ^b^	3.31 ± 0.03 ^a^

Note: Values are presented as mean ± SD. Superscript letters (e.g., a, b) within the same row represent statistically significant differences at *p* ≤ 0.05. ns indicates no significant difference (*p* > 0.05).

## Data Availability

The original contributions presented in the study are included in the article, further inquiries can be directed to the corresponding author.
